# Promising Fungicides from Allelochemicals: Synthesis of Umbelliferone Derivatives and Their Structure–Activity Relationships

**DOI:** 10.3390/molecules23113002

**Published:** 2018-11-16

**Authors:** Le Pan, Dongyu Lei, Lu Jin, Yuan He, Qingqing Yang

**Affiliations:** 1 Chemical Engineering College, Xinjiang Agricultural University, Urumqi 830052, China; hy941813@163.com (Y.H.); yqq1509914@sina.com (Q.Y.); 2 Department of Physiology, Preclinical School, Xinjiang Medical University, Urumqi 830011, China; leidongyu0109@163.com

**Keywords:** allelochemical, umbelliferone derivatives, antifungal activity, EC_50_ values

## Abstract

Umbelliferone was discovered to be an important allelochemical in our previous study, but the contribution of its activity and structure has not yet been revealed. In this study, a series of analogues were synthesized to determine the skeleton of umbelliferone and examine its fungicidal activity. Furthermore, targeted modifications were conducted with three plant parasitic fungi to examine the lead compounds. Among those tested, compounds **2f** and **10** were found to show excellent antifungal activity with an inhibitory rate over 80% at 100 ug/mL. The study proves that umbelliferone can be a promising skeleton for fungicides discovery. In addition, the primary structure–activity relationship provides a good guidance for the discovery of novel fungicides based on natural products in the future.

## 1. Introduction

Plant pathogenic fungi have jeopardized crop yields and food security globally. Fungicides have always been an efficient way of minimizing the cost of plant diseases [1]. However, the widespread use of traditional fungicides has led to increased resistance, mammalian toxicity, and a threat to the environment. This has stimulated the demand for integrated control measures with more selective, lower dose agents that have improved resistance [2,3,4,5]. Natural products (NPs) have been a rich source of novel fungicide development either with direct application or serving as a model for advanced modification [6]. However, with the explosive growth in NPs, it has become increasingly more difficult to pick the appropriate product from such a large range of compounds, and the introduction of novel molecules to the market has recently declined. Therefore, there is an urgent need for cost-effective tactics, instead of “blind search”, in the development of new fungicides [7].

**Figure 1:**
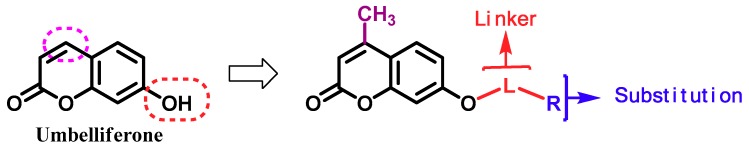
Umbelliferone and its potential modification sites.

In nature, allelochemicals play an important role in plant ecological competition by inhibiting or enhancing the growth of the target organism. Allelochemicals have therefore been proposed as a promising means in the discovery of growth regulators and agrochemicals [8,9,10]. Coumarins are widely distributed in plants, and they have been proven to play a key role in plant allelopathic effect [11,12]. In our previous study, umbelliferone was identified as an important allelochemical of *Stellera chamaejasme*, and its analogs were synthesized to investigate antifungal activity. The structure–activity relationship (SAR) indicated that the modification of umbelliferone at the C7 hydroxy not only enhanced fungicidal activity but also decreased phytotoxicity [13]. In light of the above findings, coumarin derivatives were proposed to be an optimal skeleton (Figure 1). In this study, a series of compounds were designed to confirm the possible application of umbelliferone, and C7-substituted umbelliferones derivatives (UDs) were designed and synthesized to investigate their fungicidal activity against three devastating fungi: *Alternaria alternata*, *Botrytis cinerea*, and *Fusarium oxysporum*.

## 2. Results and Discussion

**Scheme 1:**
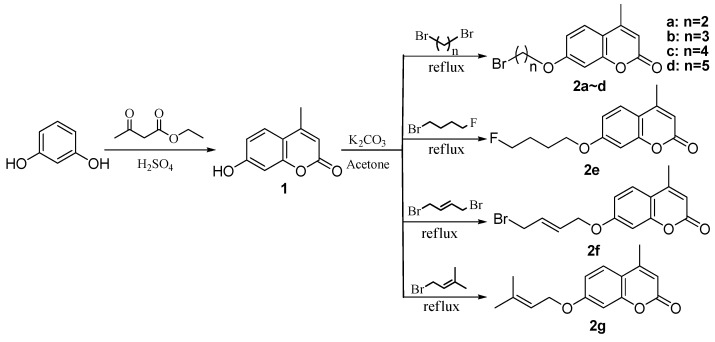
Synthesis of compounds **1**–**2g**.

**Scheme 2:**
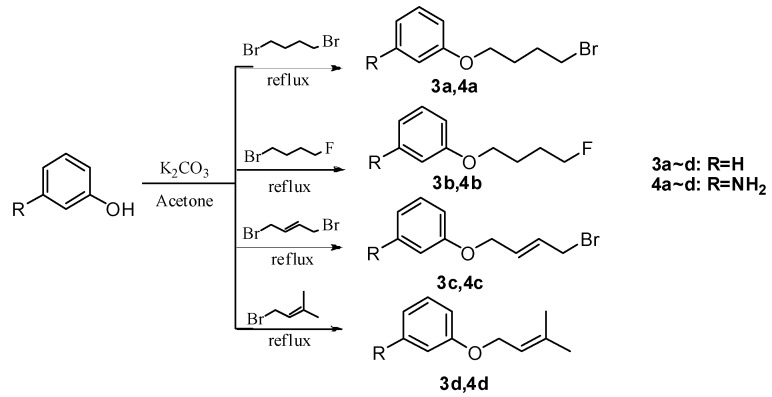
Synthesis of compounds **3a**–**4d**.

**Scheme 3:**
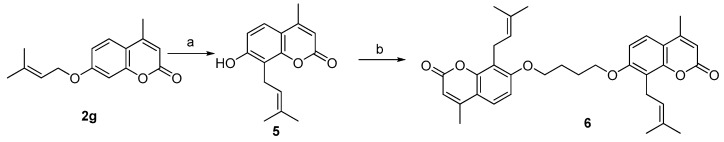
Synthesis of compounds **5** and **6**. Reagents and conditions: (**a**) 200 °C; (**b**) 1,4-dibromobutane; KOH; KI and TBAB; reflux in acetonitrile.

**Scheme 4:**
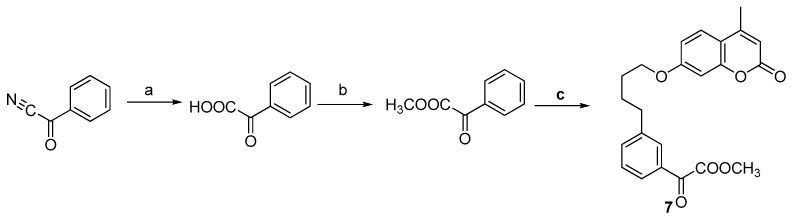
Synthesis of compound **7**. Reagents and conditions: (**a**) HCl, stirring at room temperature; (**b**) methanol; H_2_SO_4_; reflux; (**c**) compound **2c**; AlCl_3_; 80 °C.

**Scheme 5:**
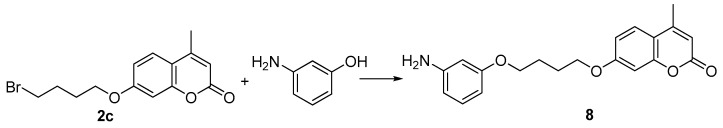
Synthesis of compound **8**. Reagents and conditions: KOH; KI and TBAB; reflux in acetonitrile.

**Scheme 6:**
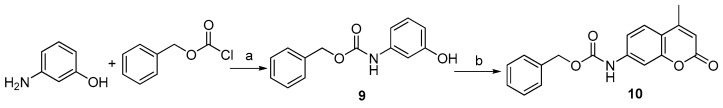
Synthesis of compound **9** and **10**. Reagents and conditions: (**a**) para-dioxane; stirred at 0 °C; (**b**) ethyl acetoacetate; phosphoric acid; 80 °C.

The preparation of umbelliferone (**1**) and 7-(4-bromoalkoxy)-4-methyl benzopyran-2-one (**2c**–**g**) was performed as per our previous methods. The rest of the UDs were synthesized according to the following method by an alkylation reaction, Friedel-Crafts reaction, and Claisen rearrangement reaction (Scheme 1, Scheme 2, Scheme 3, Scheme 4, Scheme 5 and Scheme 6) [14,15,16].

Fungicidal activity and structure-activity relationship: The fungicidal activity of the compounds is shown in Table 1. As we had predicted, 4-methylumbelliferone (**1**) and its 7-subsituted derivatives exhibited significant inhibitory effects at the concentration of 100 mg/L. In particular, compound **2f** exhibited a significant activity with inhibitory rate up to 90%, and compounds **2a**−**e** and **2g** displayed moderate to strong activity against all tested fungi. The EC_50_ values of derivatives were evaluated at a series of lower concentration, which are shown in Table 2. The results indicated that compound **2f** had the lowest EC_50_ values of 50.5 μmol/L, 30.7 μmol/L, and 58.9 μmol/L against *A. alternata*, *B. cinerea*, and *F. oxysporum*, respectively.

It is worth noting that the side chain was important to the activity exhibition. As the length of the side chain increased from two to five carbon atoms, the activity varied with nonlinear changes. It was clearly seen that four carbon atoms was optimum on activity exhibition, which was in accordance with our previous study. In order to further confirm the contribution of the coumarin skeleton, its core structure of benzopyran-2-one was substituted by benzene (**3a**–**d**) and aniline (**4a**–**d**). The activity was found to be significantly decreased compared with the related umbelliferone derivatives, except (4-bromobutoxy) benzene (**3a**). Therefore, the results proved that taking umbelliferone as the main skeleton, together with our proposed modification strategy, was evidently effective.

Based on the above findings, a series of coumarin derivatives were designed and screened for their antifungal activity. The results indicated that the functional groups played an important role in activity exhibition. Halides, especially fluoro compounds, are widely applied in lead compound modification and have been proven to show good biocompatibility and activity [17]. In this study, it was found that the inhibition rate increased significantly with compound **2e** (about 65%) compared to compound **1** (about 30%) when the terminal bromine was substituted with fluorine. We also deemed it meaningful to introduce different fluoro moieties in our studies. Isopentenyl has been reported to play an important role in membrane binding and activity expression of biomolecules [18,19]. Our results showed similar results, with activity being enhanced when isopentenyl was introduced as a terminal moiety. Methoxyacrylate, originated from strobilurin A, is a key skeleton in commercial fungicides [20,21]. By coupling methoxyacrylate with coumarin with four carbon chain (**7**), we managed to achieve good activity. The type of linkage also played an important role in antifungi activity. The results indicated that the inhibitory effect of compound **2f** was much better than compound **2c** due to the slight structural difference between the two, with compound **2f** having an olefinic linkage instead of the alkoxy side chain of compound **2c**. We propose that the increase in activity may have been induced by the conjugation of double bond, O atom, and aromatic ring in compound **2f**. 

## 3. Experimental Section

All starting chemicals were purchased from commercial vendors, were of AR grade, and were used without purification. Reactions were monitored by thin-layer chromatography (Silica Gel 60 F254). Target compounds were purified with column chromatography using silica gel (200–300 mesh). ^1^H-NMR (400 MHz) and ^13^C-NMR (100 MHz) spectra were recorded on a Bruker AM-400BB spectrometer (Bruker, Karlsruhe, Germany) in CDCl_3_/DMSO-*d*_6_ solution with TMS as the internal standard. The chemical shift values (δ) below are listed in ppm and the coupling constant values (*J*) are in Hertz. The melting points were determined with a X-4 melting point apparatus. The purity of all test compounds was above 95%. Compounds **2a** and **2b** were prepared as per our previous study and used directly [13].

### 3.1. General Procedure for the Preparation of Compounds ***2c**–**g***

1,4-Dibromobutane, 1,5-dibromopentane, 1-bromo-4-fluorobutane, (2E)-1,4-dibromo-2-butene (3.0 mmol), or 1-bromo-3-methyl-2-butene (5.0 mmol) was added to a solution of 4-methylcoumarin (3.5 mmol), KOH (1 mmol), and K_2_CO_3_ (4 mmol) in acetone (20 mL), respectively. The reaction was heated at reflux detected with TLC for 5 h. Then, the crude products were prepared by filtering the mixture and evaporating the solvent and were further purified by silica chromatography (CHCl_3_/MeOH 50/1) to give compounds **2c**–**g** (Scheme 1).

*7-(4-Bromobutoxy)-4-methylbenzopyran-2-one* (**2c**): White solid; yield: 65%; m.p., 42–44 °C; ESI-MS *m*/*z*: 312.2 [M + H]^+^. ^1^H-NMR (CHCl_3_-d, 400 MHz) δ:1.98–2.02 (m, 2H), 2.06–2.12 (m, 2H), 2.40 (s, 3H), 3.50 (t, 2H, *J* = 8.0Hz), 4.06 (t, 2H, *J* = 8.0 Hz), 6.14 (s, 1H), 6.80 (d, 1H, *J* = 4.0 Hz), 6.82 (dd, 1H, *J* = 8.0 Hz), 7.49 (d, 1H, *J* = 8.0 Hz); ^13^C-NMR (CHCl_3_-d, 100 MHz) δ: 27.69, 29.34, 32.16, 67.56, 101.41, 112.61, 112.87, 113.18, 128.79, 143.36, 155.93, 161.15, 162.09.

*7-(4-Bromopentyloxy)-4-methylbenzopyran-2-one* (**2d**): White solid; yield: 60%; m.p., 60–62 °C; ESI-MS *m*/*z*: 326.2 [M + H]^+^. ^1^H-NMR (CHCl_3_-d, 400 MHz) δ:1.30–1.34 (m, 2H), 1.92–1.98 (m, 2H), 2.02–2.06 (m, 2H), 2.42 (s, 3H), 3.51 (t, 2H, *J* = 8.0 Hz), 4.08 (t, 2H, *J* = 8.0 Hz), 6.15 (s, 1H), 6.82 (d, 1H, *J* = 2.4 Hz), 6.88 (dd, 1H, *J* = 8.4 Hz, 2.4 Hz), 7.50 (d, 1H, *J* = 8.4Hz).

*7-(4-Fluorobutoxy)-4-methylbenzopyran-2-one* (**2e**): White solid; yield: 63%; m.p., 116–118 °C; ESI-MS *m*/*z*: 251.3 [M + H]^+^. ^1^H-NMR (CHCl_3_-d, 400 MHz) δ: 1.86–1.98 (m, 4H), 2.40 (s, 3H), 4.07 (t, 2H, *J* = 8.0 Hz), 4.79 (t, 2H, *J* = 8.0 Hz), 6.13 (s, 1H), 6.80 (d, 1H, *J* = 2.4 Hz), 6.85 (dd, 1H, *J* = 8.8 Hz, 2.4 Hz), 7.49 (d, 1H, *J* = 8.8Hz).

*7-(4-Bromobutenyloxy)-4-methylbenzopyran-2-one* (**2f**): White solid; yield: 65%; m.p., 116–118 °C ESI-MS *m*/*z*: 310.2 [M + H]^+^. ^1^H-NMR (CHCl_3_-d, 400 MHz) δ: 2.40 (s, 3H), 4.00 (d, 2H, *J* = 7.2 Hz), 4.62 (d, 2H, *J* = 4.8 Hz), 6.01–6.15 (m, 2H), 6.15 (s, 1H), 6.80 (d, 1H, *J* = 2.0 Hz), 6.87 (dd, 1H, *J* = 8.8 Hz, 2.4 Hz), 7.55 (d, 1H, *J* = 8.8 Hz).

*7-(4-Isopentenyloxy)-4-methylbenzopyran-2-one* (**2g**): White solid; yield: 75%; ESI-MS *m*/*z*: 247.3 [M + H]^+^. ^1^H-NMR (CHCl_3_-d, 400 MHz) δ:1.77 (s, 3H), 1.81 (s, 3H), 2.49 (s, 3H), 4.58 (d, 2H, *J* = 8.0 Hz), 5.47–5.50 (m, 1H), 6.13 (s, 1H), 6.82 (d, 1H, *J* = 2.0 Hz), 6.87 (dd, 1H, *J* = 8.8Hz, *J* = 2.4Hz), 7.49 (d, 1H, *J* = 8.8Hz).

### 3.2. General Procedure for the Preparation of Compounds ***3a**–**d*** and ***4a**–**d***

1,4-Dibromobutane, 1-bromo-4-fluorobutane, (2E)-1,4-dibromo-2-butene or 1-bromo-3-methyl-2-butene(3.0 mmol) was added to a solution of benzene (**3a**–**d**) or aniline (**4a**–**d**) (3.5 mmol), KOH (1 mmol), and K_2_CO_3_ (4 mmol) in acetone (20 mL), respectively. The reaction was heated at reflux detected with TLC. Then, the crude products were further purified by silica chromatography (CHCl_3_/MeOH 50/1) to give compounds **3a**–**d** and **4a**–**d** (Scheme 2).

*(4-Bromobutoxy) benzene* (**3a**): White oil; yield: 75%; m.p., 40–41 °C; ^1^H-NMR (CHCl_3_-d, 400 MHz) δ:1.74–1.78 (m, 2H), 1.80–1.86 (m, 2H), 3.51 (t, 2H, *J* = 8.0 Hz), 4.08 (t, 2H, *J* = 8.0 Hz), 6.99 (dd, 2H, *J* = 8.0 Hz, 2.4 Hz), 7.03 (dd, 1H, *J* = 8.0 Hz, 2.4 Hz), 7.37–7.40 (m, 2H). 

*(4-Fluorobutoxy) benzene* (**3b**): White oil; yield: 65%; m.p., 55–57 °C; ^1^H-NMR (CHCl_3_-d, 400 MHz) δ: 1.70–1.86 (m, 4H), 4.07 (t, 2H, *J* = 8.0 Hz), 4.50 (t, 2H, *J* = 8.0 Hz), 6.96 (dd, 2H, *J* = 8.0 Hz, 2.4 Hz), 7.02(dd, 1H, *J* = 8.0 Hz, 2.4 Hz), 7.33–7.36 (m, 2H).

*7-(4-Bromobutenyloxy) benzene* (**3c**): White solid; yield: 72%; m.p., 46–48 °C; ^1^H-NMR (CHCl_3_-d, 400 MHz) δ: 4.02 (d, 2H, *J* = 7.2 Hz), 4.65 (d, 2H, *J* = 4.8 Hz), 6.00–6.12(m, 2H), 7.03 (dd, 1H, *J* = 8.8 Hz, *J* = 2.0 Hz), 7.05 (dd, 2H, *J* = 8.8 Hz, *J* = 2.0 Hz), 7.34–7.38 (m, 1H).

*(4-Isopentenyloxy) benzene* (**3d**): Yellow oil; yield: 76%; ^1^H-NMR (CHCl_3_-d, 400 MHz) δ: 1.75 (s, 3H), 1.80 (s, 3H), 4.59 (d, 1H, *J* = 8.0 Hz), 5.46 (m, 1H), 7.02 (dd, 1H, *J* = 8.8 Hz, *J* = 2.4Hz), 7.04 (dd, 2H, *J* = 8.8 Hz, *J* = 2.4Hz), 7.34–7.37 (m, 2H).

*(4-Bromobutoxy) aniline* (**4a**): White solid; yield: 63%; m.p., 116–118 °C; ^1^H-NMR (CHCl_3_-d, 400 MHz) δ:1.74–1.82 (m, 4H), 3.52 (t, 2H, *J* = 8.0 Hz), 3.68 (s, 2H), 4.09 (t, 2H, *J* = 8.0 Hz), 6.16–6.19 (m, 2H), 6.35 (dd, 1H, *J* = 8.0 Hz, 2.4 Hz), 7.09 (dd, 1H, *J* = 8.4 Hz, 8.2 Hz).

*(4-Fluorobutoxy) aniline* (**4b**): Yellow solid; yield: 60%; m.p., 76–78 °C; ^1^H-NMR (CHCl_3_-d, 400 MHz) δ: 1.62–1.78 (m, 4H), 3.66 (s, 2H), 4.06 (t, 2H, *J* = 8.0 Hz), 4.52 (t, 2H, *J* = 8.0 Hz), 6.21 (dd, 1H, *J* = 8.0 Hz, 2.4 Hz), 6.25 (dd, 1H, *J* = 2.4 Hz, 2.0 Hz), 6.28–6.31 (m, 1H), 7.02 (dd, 1H, *J* = 8.4 Hz, 8.0 Hz).

*(4-Bromobutenyloxy) aniline* (**4c**): Yellow solid; m.p., 121–123 °C; ^1^H-NMR (CHCl_3_-d, 400 MHz) δ: 3.62 (s, 2H), 4.02 (d, 2H, *J* = 7.2 Hz), 4.68 (d, 2H, *J* = 4.8 Hz), 6.00–6.12 (m, 2H), 6.16–6.20 (m, 1H), 6.22 (dd, 1H, *J* = 2.4 Hz, 2.0 Hz), 6.38 (m, 1H), 7.05 (dd, 1H, *J* = 8.4 Hz, 8.0 Hz).

*(4-Isopentenyloxy) aniline* (**4d**): Yellow solid; yield: 68%; m.p.,68–70 °C; ^1^H-NMR (CHCl_3_-d, 400 MHz) δ: 1.72 (s, 3H), 1.84 (s, 3H), 3.70 (s, 2H), 4.56 (d, 1H, *J* = 8.0 Hz), 5.44 (m, 1H), 6.14–6.21 (m, 1H), 6.23 (dd, 1H, *J* = 8.4 Hz, 2.0 Hz), 6.35–6.41 (m, 1H), 7.07 (dd, 1H, *J* = 8.4 Hz, 8.0 Hz).

### 3.3. General Procedure for the Preparation of Compounds ***5*** and ***6***

Compound **2g** was added into single-necked flask and heated to 200 °C. Then, the mixture was dissolved into methanol and purified by silica chromatography to get compound **5**. Then, compound **5** (1.5 mmol) was added to a solution of 1, 4-dibromobutane (2 mmol), KOH (1 mmol), and K_2_CO_3_ (2 mmol) in acetone (10 mL). The reaction was heated at reflux for 3 h. The crude products were prepared by filtering the mixture and evaporating the solvent and were further purified by silica chromatography to give compound **6**.

*7-Hydroxy-6-isopentenyl-4-methylbenzopyran-2-one* (**5**): White solid; yield: 30%; m.p., 83–84 °C; ESI-MS *m*/*z*: 245.3 [M + H]^+^. ^1^H-NMR (CHCl_3_-d, 400 MHz) δ: 1.70 (s, 3H), 1.83 (s, 3H), 2.41 (s, 3H), 3.30 (d, 2H, *J* = 6.0 Hz), 4.08 (t, 1H, *J* = 6.0 Hz), 6.09 (s, 1H), 6.69 (d, 1H, *J* = 2.0 Hz), 6.79–6.82 (dd, 1H, *J* = 8.8 Hz, *J* = 2.4Hz), 7.58 (d, 1H, *J* = 8.8 Hz); ^13^C-NMR (CHCl_3_-d, 100 MHz) δ: 17.24, 19.34, 22.4, 24.62, 102.06, 109.82, 112.44, 112.94, 115.1, 125.20, 126.03 154.56, 155.15, 161.63, 162.46.

*1,4-Di(6-isopentenyl-4-methylbenzopyran-2-one-7-oxy)butane* (**6**): White solid; yield: 20%; m.p.,162–164 °C; ESI-MS *m*/*z*: 529.6 [M + H]^+^. ^1^H-NMR (CHCl_3_-d, 400 MHz) δ:1.28 (s, 6H), 1.35 (s, 6H), 2.03–2.07 (m, 4H), 2.44 (s, 6H, *J* = 6.0 Hz), 3.26 (d, 4H, *J* = 6.0 Hz), 4.05 (d, 2H, *J* = 8.0 Hz), 4.60 (d, 2H, *J* = 8.0 Hz), 6.06 (s, 2H), 6.16 (s, 2H), 6.90 (d, 2H, *J* = 8.8 Hz), 6.98 (dd, 2H, *J* = 8.8 Hz, *J* = 2.0 Hz), 7.68 (d, 2H, *J* = 9.6 Hz); ^13^C-NMR (CHCl_3_-d, 100 MHz) δ: 17.25, 19.11, 19.33, 24.92, 30.68, 63.69, 67.78, 101.25, 112.69, 125.95, 127.62, 128.17, 128.98, 129.84, 154.29, 155.00, 162.11

### 3.4. General Procedure for the Preparation of Compound ***7***

Benzoylcyanide (3 mmol) was dissolved in hydrochloric acid (10 mL) with stirring at room temperature for 2h. Then, the products were filtered out and added into methanol. Concentrated H_2_SO_4_ (1 mL) was added to the mixture and refluxed. After that, the mixture was extracted with ethyl acetate and evaporated to yield the crude products, which reacted with compound **2c** without purification catalyzed by AlCl_3_. The crude products were further purified by means of silica chromatography to provide compound **7** (Scheme 2).

*1-(4-Methylbenzopyran-2-one-7-oxy)-4-(3-methylphenylglyoxylate)butane* (**7**): White solid; yield: 30%; m.p.,117–119 °C; ESI-MS *m*/*z*: 395.4 [M + H]^+^. ^1^H-NMR (CHCl_3_-d, 400 MHz) δ: 1.22–1.27 (m, 2H), 1.71–1.79 (m, 2H), 2.40 (s, 3H), 2.86–2.91 (m, 2H), 3.68 (s, 3H), 4.14–4.18 (m, 2H), 6.16 (s, 1H), 6.85 (dd, 1H, *J* = 8.4 Hz, 2.4 Hz), 7.48 (t, 1H, *J* = 8.4 Hz), 7.57(dd, 1H, *J* = 8.0 Hz, 2.4 Hz), 7.62 (t, 1H, *J* = 8.4 Hz), 7.70–7.73 (m, 2H), 8.11(d, 1H, *J* = 8.0 Hz); ^13^C-NMR (CHCl_3_-d, 100 MHz) δ: 18.73, 22.73, 29.71, 31.68, 52.34, 68.70, 103.39, 111.63, 112.50, 113.07, 113.57, 125.41, 125.96, 128.51, 130.20, 133.72, 145.2, 152.7, 153.15, 155.12, 159.71, 161.89, 170.43.

### 3.5. General Procedure for the Preparation of Compound ***8***

Compound **2c** (2.0 mmol) was added to a solution of 3-aminophenol (2.5 mmol), KOH (1 mmol), and K_2_CO_3_ (3 mmol) in acetone (10 mL). The mixture was heated at reflux for 3 h and the reaction was detected with TLC. The mixture was extracted with ethyl acetate and evaporated to get crude product. The crude products were prepared by filtering the mixture and evaporating the solvent and were further purified by silica chromatography to give compound **8** (Scheme 3).

*1-(4-Methylbenzopyran-2-one-7-oxy)-4-(3-aminophenol) butane* (**8**): White solid; yield: 54%; m.p., 123–125 °C; ESI-MS *m*/*z*: 340.4 [M + H]^+^. ^1^H-NMR (CHCl_3_-d, 400 MHz) δ: 1.23–1.28 (m, 2H), 2.02–2.10 (m, 2H), 2.40 (s, 3H), 4.11 (s, 2H), 4.51–4.57 (m, 2H), 4.62–4.66 (m, 2H), 6.02–6.09 (m, 1H), 6.14 (s, 1H), 6.19–6.34 (m, 2H), 6.85–6.89 (m, 1H), 7.05 (t, 1H, *J* = 8.4 Hz), 7.48 (t, 2H, *J* = 8.8 Hz); ^13^C-NMR (CHCl_3_-d, 100 MHz) δ: 18.69, 27.8(2), 67.32(2), 101.74, 104.69, 108.29, 112.08, 113.73, 126.88, 128.43, 129.56, 130.16, 147.81, 152.59, 155.12, 159.71, 161.34, 161.50.

### 3.6. General Procedure for the Preparation of Compounds ***9*** and ***10***

The amine (5.0 mmol) was dissolved in water, and NaHCO_3_ (10.0 mmol) was added with stirring. After being cooled to 5 °C, Cbz-Cl (7.5 mmol) was added slowly in para-dioxane. Then, the mixture was stirred at 0 °C for 1 h and allowed to warm to room temperature for 12 h. After the reaction, water was added and extracted with ethyl acetate. The aqueous layers were acidified to pH of 1 and extracted three times with ethyl acetate. The organic layers were dried with sodium sulfate, and the solvent was evaporated to get compound **9**. Ethyl acetoacetate (2 mmol) was added to a mixture of compound **9** (1 mmol) and phosphoric acid (10 mL). The mixture was heated with stirring at 80 °C for 5 h. After cooling down, the mixture was quenched with ice-water. The crude product was filtered out, dried, and purified by recrystallization from ethanol to yield compound **10** (Scheme 4).

*N-Carbobenzoxy-3-hydroxyaniline* (**9**): White solid; yield: 68%; ESI-MS *m*/*z*: 244.3 [M + H]^+^. m.p., 204–206 °C; ^1^H-NMR (CHCl_3_-d, 400 MHz) δ: 4.62 (s, 2H), 5.35 (s, 1H), 6.65 (dd, 1H, *J* = 8.0, Hz, 2.0 Hz), 7.12 (dd, 1H, *J* = 8.0 Hz, 2.0 Hz), 7.25–7.38 (m, 5H), 7.47 (dd, 2H, *J* = 8.0 Hz, 2.0 Hz), 9.10 (s, 1H); ^13^C-NMR (CHCl_3_-d, 100 MHz) δ: 68.76, 105.10, 113.42, 114.18, 127.26(2), 127.60, 128.92(2), 130.20, 136.15, 137.30, 153.38, 158.78.

*4-Methyl-7-(N-carbobenzoxy)-aminocoumarin* (**10**): White solid; yield: 28%; m.p., 228–230 °C; ESI-MS *m*/*z*: 310.4 [M + H]^+^. ^1^H-NMR (CHCl_3_-d, 400 MHz) δ: 2.40 (s, 3H), 5.23 (s, 2H), 6.19 (s, 1H), 6.97 (s, 1H), 7.37–7.42 (m, 5H), 7.44 (d, 1H, *J* = 1.6 Hz), 7.52 (d, 2H, *J* = 8.4 Hz); ^13^C-NMR (CHCl_3_-d, 100 MHz) δ: 18.69, 67.62, 106.96, 113.24, 114.36, 115.60, 125.39, 128.45(2), 128.59, 128.72 (2), 135.57, 141.32, 152.19, 152.77, 154.58, 161.06.

### 3.7. Antifungal Activity 

The fungicidal activities were investigated with three plant parasitic fungi (*Alternaria alternata*, *Botrytis cinerea*, and *Fusarium oxysporum*), and mycelial inhibition of radial growth on PDA media was employed [22,23].

## 4. Conclusions

In summary, the umbelliferone skeleton was revealed to play a key role in the antifungal activity. The structure–activity relationship study showed that coumarin coupled with haloalkane, methoxyacrylate, and isopentenyl moieties could significantly enhance activity. The research provides a good guidance for further study of the molecular design and discovery of allelochemical-based agents for phytopathogenic fungi control.

## Figures and Tables

**Table 1:** In vitro fungicidal activities of the prepared compounds at 100 ug/mL (96 h of incubation).

Compd.	The Inhibition Rate (%; mean ± SD; N = 3)		
	*A. Alternata*	*B. Cinerea*	*F. Oxysporum*
**1**	38.5 ± 4.38	25.8 ± 3.8	41.2 ± 2.5
**2a**	38.1 ± 5.9	39.6 ± 6.2	33.2 ± 4.4
**2b**	42.9 ± 6.7	36.3 ± 5.8	28.6 ± 3.4
**2c**	70.6 ± 5.5	68.3 ± 4.6	60.4 ± 5.3
**2d**	40.3 ± 3.7	46.2 ± 5.8	35.5 ± 7.2
**2e**	63.7 ± 3.9	72.9 ± 3.5	62.8 ± 5.6
**2f**	81.5 ± 3.7	90.99 ± 2.3	84.8 ± 3.2
**2g**	48.4 ± 4.0	70.6 ± 5.2	53.5 ± 4.2
**3a**	49.6 ± 3.6	41.2 ± 5.4	50.2 ± 6.3
**3b**	3.8 ± 2.8	10.2 ± 2.6	8.4 ± 4.1
**3c**	58.2 ± 3.6	63.0 ± 7.0	61.3 ± 3.8
**3d**	29.6 ± 5.3	32.5 ± 6.2	26.2 ± 4.9
**4a**	30.5 ± 5.2	32.1 ± 4.5	28.4 ± 5.8
**4b**	35.3 ± 5.6	46.2 ± 3.7	33.2 ± 3.1
**4c**	12.6 ± 2.6	15..0 ± 6.4	13.7 ± 4.2
**4d**	10.2 ± 3.5	12.6 ± 4.5	9.8 ± 2.6
**5**	22.4 ± 4.7	26.4 ± 2.1	32.6 ± 5.6
**6**	27.6 ± 8.3	35.93 ± 1.73	29.8 ± 3.8
**7**	20.8 ± 7.7	21.9 ± 3.0	18.7 ± 4.1
**8**	26.6 ± 3.9	18.9 ± 8.5	25.3 ± 5.2
**9**	58.5 ± 3.4	70.9 ± 1.7	77.5 ± 3.4
**10**	77.3 ± 5.5	84.4 ± 3.5	80.5 ± 5.7
**DMSO (1%)**	0	0	0
**carbendazim**	90.7 ± 8.18	94.3 ± 6.4	91.4 ± 5.4

**Table 2:** EC_50_ values and log P of effective compounds against three phytopathogenic fungi.

Compound	Log P	EC_50_ (95% CI μmol/L)		
		*A. Alternata*	*B. Cinerea*	*F. Oxysporum*
**1**	1.61	922.4 (827.6–1098.9)	1037.1 (955.3–1201.1)	1165.9 (1076.2–1268.1)
**2c**	3.26	193.5 (145.6–258.7)	221.1 (137.2–270.9)	245.2 (180.9–298.2)
**2e**	2.62	381.6 (282.1–466.7)	360.4 (274.1–462.7)	405.9 (311.3–505.9)
**2f**	3.22	50.5 (28.5–82.2)	30.7 (15.5–47.5)	58.9 (30.4–89.3)
**2g**	3.11	232.1 (158.8–336.9)	280.8(206.7–350.4)	207.1 (149.8–279.2)
**9**	2.87	185.8 (117.6–254.0)	130.7 (76.0–195.7)	182.9 (145.9–248.2)
**10**	3.23	105.1 (69.5–176.2)	66.6 (50.7–115.1)	60.8 (34.6–120.6)
**Carbendazim**	1.29	18.8 (6.8–30.0)	20.6 (7.8–32.4)	33.5 (14.6–49.7)
